# Supplementary material for: Identification of photoperiod-induced specific miRNAs in the adrenal glands of Sunite sheep (Ovis aries)
Source: Front Vet Sci. 2022 Jul 22;9:888207. doi: 10.3389/fvets.2022.888207 (PMC9354845; doi:10.3389/fvets.2022.888207)
Supplement: Supplementary file 1 [file Data_Sheet_1.ZIP › Supplementary file/miRDeep2 file.zip!/miRDeep2 file/Know_Structure/SP42A1/oar-mir-22.pdf]

miRBase precursor : oar-mir-22
Total read count : 89415
oar-miR-22-3p read count : 84606
remaining reads : 4809

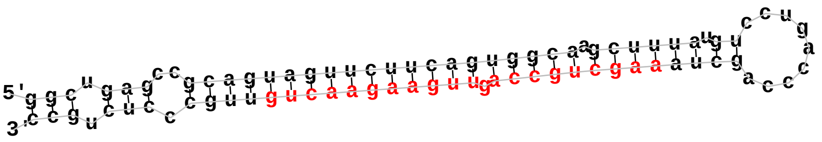

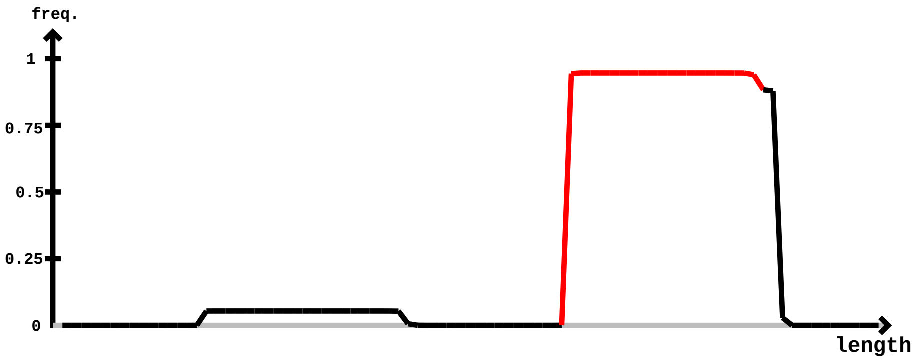

oar-miR-22-3p

```
5'- ggcugagccgcaguaguucuucaguggcaagcuuuauguccugacccagcuaaagcugccaguugaagaacuguugcccucugcc   -3'    exp
    (((.(((..((((((((((((((((((((.((((((.((.........))))))))))))).))))))))))))))).))).)))    reads      mm        sample
    .............uaguucuucaguggcaagcuuu..................................................    9          0         seq
    ..............aguucuucaguggcaagU.....................................................    1          1         seq
    ..............aguucuucaguggcaagcu....................................................    2          0         seq
    ..............aguucuucaguggcaagcA....................................................    1          1         seq
    ..............aguucuucaguggcaagcuu...................................................    38         0         seq
    ..............aguucuucaguggcaagcuuu..................................................    4224       0         seq
    ..............aguucuucaguggcaagcuuua.................................................    496        0         seq
    ..............aguucuucaguggcaagcuuuau................................................    28         0         seq
    ...............guucuucaguggcaagcuuu..................................................    8          0         seq
    ...............guucuucaguggcaagcuuua.................................................    2          0         seq
    ...................................................aaagcugccaguugaagaacu.............    7          0         seq
    ...................................................aaagcugccaguugaagaacugu...........    16         0         seq
    ....................................................aagcugccaguugaaga................    11         0         seq
    ....................................................aagcugccaguugaagaa...............    12         0         seq
    ....................................................aagcugccaguugaagaac..............    564        0         seq
    ....................................................aagcugccaguugaagaacu.............    5032       0         seq
    ....................................................aagcugccaguugaagaacug............    358        0         seq
    ....................................................aagcugccaguugaagaacugu...........    75879      0         seq
    ....................................................aagcugccaguugaagaacuguu..........    2515       0         seq
    ....................................................aagcugccaguugaagaacuguug.........    2          0         seq
    .....................................................agcugccaguugaagaac..............    2          0         seq
    .....................................................agcugccaguugaagaacu.............    13         0         seq
    .....................................................agcugccaguugaagaacugu...........    180        0         seq
    .....................................................agcugccaguugaagaacuguu..........    11         0         seq
    ......................................................gcugccaguugaagaacugu...........    2          0         seq
    ......................................................gcugccaguugaagaacuguu..........    1          0         seq
    .......................................................cugccaguugaagaacuguu..........    1          0         seq
```
